# Delivery of maternal health care in Indigenous primary care services: baseline data for an ongoing quality improvement initiative

**DOI:** 10.1186/1471-2393-11-16

**Published:** 2011-03-07

**Authors:** Alice R Rumbold, Ross S Bailie, Damin Si, Michelle C Dowden, Catherine M Kennedy, Rhonda J Cox, Lynette O'Donoghue, Helen E Liddle, Ru K Kwedza, Sandra C Thompson, Hugh P Burke, Alex DH Brown, Tarun Weeramanthri, Christine M Connors

**Affiliations:** 1 Discipline of Obstetrics and Gynaecology, The University of Adelaide, Adelaide, SA, Australia; 2 Menzies School of Health Research, Darwin, NT, Australia; 3 Centre for Chronic Disease, The University of Queensland, Brisbane, QLD, Australia; 4 Ngalkanbuy Health Service, Elcho Island, NT, Australia; 5 Marri Ma Health Aboriginal Corporation, Broken Hill, NSW, Australia; 6 Combined Universities Centre for Rural Health, University of Western Australia; 7 Menzies School of Health Research, Alice Springs, NT, Australia; 8 Queensland Health, Brisbane, QLD, Australia; 9 Centre for Indigenous Vascular and Diabetes Research, Baker IDI Heart and Diabetes Institute, Alice Springs, NT, Australia; 10 Public Health Division, Department of Health, Government of Western Australia, Perth, WA, Australia; 11 Northern Territory Department of Health and Families, Darwin, NT, Australia

## Abstract

**Background:**

Australia's Aboriginal and Torres Strait Islander (Indigenous) populations have disproportionately high rates of adverse perinatal outcomes relative to other Australians. Poorer access to good quality maternal health care is a key driver of this disparity. The aim of this study was to describe patterns of delivery of maternity care and service gaps in primary care services in Australian Indigenous communities.

**Methods:**

We undertook a cross-sectional baseline audit for a quality improvement intervention. Medical records of 535 women from 34 Indigenous community health centres in five regions (Top End of Northern Territory 13, Central Australia 2, Far West New South Wales 6, Western Australia 9, and North Queensland 4) were audited. The main outcome measures included: adherence to recommended protocols and procedures in the antenatal and postnatal periods including: clinical, laboratory and ultrasound investigations; screening for gestational diabetes and Group B Streptococcus; brief intervention/advice on health-related behaviours and risks; and follow up of identified health problems.

**Results:**

The proportion of women presenting for their first antenatal visit in the first trimester ranged from 34% to 49% between regions; consequently, documentation of care early in pregnancy was poor. Overall, documentation of routine antenatal investigations and brief interventions/advice regarding health behaviours varied, and generally indicated that these services were underutilised. For example, 46% of known smokers received smoking cessation advice/counselling; 52% of all women received antenatal education and 51% had investigation for gestational diabetes. Overall, there was relatively good documentation of follow up of identified problems related to hypertension or diabetes, with over 70% of identified women being referred to a GP/Obstetrician.

**Conclusion:**

Participating services had both strengths and weaknesses in the delivery of maternal health care. Increasing access to evidence-based screening and health information (most notably around smoking cessation) were consistently identified as opportunities for improvement across services.

## Background

Approximately four percent of women giving birth in Australia are Aboriginal and/or Torres Strait Islander (Indigenous) [1]. Disproportionately high rates of poor pregnancy outcomes including maternal and perinatal mortality, preterm birth and low birth weight have consistently been documented in Indigenous populations relative to other Australians [2]. With the exception of perinatal mortality, little improvement in these outcomes has occurred in the past two decades [1], and for some outcomes (e.g. low birth weight), the disparity appears to be widening [3].

Providing access to appropriate and quality care in the antenatal and postnatal period is a key part of closing the gap in Indigenous perinatal outcomes. It also offers an important opportunity to ameliorate disease in adult life, given the recognised link between early life influences and chronic disease [4].

Antenatal care aims to provide appropriate screening, preventive or treatment interventions and health information to maximise the health of women and their infants [5]. Almost all pregnant Australian women have some antenatal care, which can be delivered by a diverse range of providers (e.g. midwives, obstetricians, Aboriginal health workers, general practitioners etc.) working across hospital and community settings. However, Indigenous women access care differently to other Australian women. Although there is no complete national data for Indigenous women, the available data suggests that antenatal care is underutilised, as Indigenous women are more likely to present for care later in pregnancy and have fewer antenatal visits [6-8].

The importance of achieving equity in access to antenatal care is recognised in several local and international development initiatives [9,10]. However, improving the quality of care is also important, as poor quality care can contribute to poor outcomes [11] and be a barrier to service utilisation [12]. Previous research assessing care for Aboriginal women birthing in the Northern Territory (NT) identified the need to focus on accurate assessment and follow up of problems identified in pregnancy, and other aspects of quality of care, rather than focusing solely on the timing and frequency of antenatal visits [13]. Furthermore, there is evidence of sustained improvements in perinatal outcomes amongst women attending a large Aboriginal Medical Service in Queensland that focussed on quality improvement in maternal and child health [14,15].

Recognising the potential benefits of quality improvement principles in primary health care, the Australian Government launched the Healthy for Life program in 2005 [16]. The program aimed to improve the capacity of and care offered by Aboriginal primary health care services for chronic disease and maternal and child health, and drew extensively from a quality improvement action research project that has been operating since 2002 - the Audit and Best practice for Chronic Disease (ABCD) Project [17]. Here we describe patterns of delivery of maternal health services and gaps in these services drawing on data from this project.

## Methods

The data presented here arise from baseline clinical audits undertaken in the ABCD Project. Of 56 participating primary health care centres in early 2009, 34 elected to audit their maternal health records. These centres were located in: a) remote communities in the Top End (13) and Central Australia (2) of the NT and in North Queensland (QLD) (4); and b) small regional towns in Far West New South Wales (NSW) (6) and Western Australia (WA) (9). Participating services were widely distributed across Australia and many of the remote health centres were located in sparsely populated areas in the northern part of Australia.

Delivery of maternal health care was assessed by auditing a sample of clinical records from participating services. Records of women with an infant aged between 2-14 months and who were resident in the community for at least six months of that infant's gestation were eligible. From eligible records in each service, a random sample of up to 30 records was drawn using computer generated random numbers. Where there were fewer than 30 eligible records all were included for audit. In the ACBD project these sampling methods have been demonstrated to be sufficient to show meaningful differences between communities and trends over time.

The audit was based on previous research [18], published antenatal guidelines [19], the Women's Business Manual (WBM) [20] and key literature [21-24]. The WBM is the standard management manual for practitioners caring for women in remote areas.

The audits were conducted by trained members of the project team familiar with the auditing process and the maternal audit tool, in collaboration with local health staff. The audit form and protocol are available at: <http://www.abcdproject.org.au >. A clinical service related to antenatal/postnatal care was assessed as "delivered" if there was a record of the service being delivered at specific periods in line with antenatal/postnatal care guidelines.

We have previously published a short report on selected data from this study [25]. Here we present a more complete analysis of the data.

The study was approved by the human research ethics committees in the Top End of the NT, Central Australia, Far West NSW, WA and Cairns and Townsville, and their Indigenous sub-committees where required.

## Results

The records of 535 women (NT Top End 136, Central Australia 45, Far West NSW 103, WA 193, and North QLD 58) from the 34 participating services were audited (Table 1). Most (59%) services were managed by a local or regional Indigenous committee/board, 38% were accredited practices and half served communities with 1000 members or more.

**Table 1:** Characteristics of participating health centres, women and early pregnancy care

Characteristic	NT Top End	NT Central Australia	Far West NSW	WA	North QLD	Total
**No. of health centres**	13	2	6	9	4	34
Governance models of health centres						
Government funded/operated	7 (54%)	0 (0%)	0 (0%)	3 (33%)	4 (100%)	14 (41%)
Managed by local or regional Indigenous committee or board	6 (46%)	2 (100%)	6 (100%)	6 (67%)	0 (0%)	20 (59%)
Accreditation status						
Accredited	6 (46%)	2 (100%)	0 (0%)	4 (44%)	1 (25%)	13 (38%)
Not accredited	7 (54%)	0 (0%)	6 (100%)	5 (56%)	3 (75%)	21 (62%)
Population sizes of communities served						
≤ 500	4 (31%)	0 (0%)	1 (17%)	0 (0%)	1 (25%)	6 (18%)
501-999	4 (31%)	1 (50%)	3 (50%)	1 (11%)	2 (50%)	11 (32%)
≥1000	5 (38%)	1 (50%)	2 (33%)	8 (89%)	1 (25%)	17 (50%)
**No. of women participating in maternal health audits**	136	45	103	193	58	535
Median age of women (years)	23.7	24.8	25.8	26.2	26.4	25.3
Indigenous status						
Indigenous	96%	100%	61%	93%	95%	89%*
Non-Indigenous	3%	0%	34%	6%	0%	9%*
Not stated	1%	0%	5%	1%	5%	2%
Mean estimated gestational age at the first antenatal visit (weeks)	14	15	20	15	15	16
Proportion of women with estimated gestational age at first antenatal visit < 12 weeks	49%	44%	35%	42%	34%	42%
Mean number of antenatal visits	9	10	5	6	7	7*
First antenatal assessments by:						
Aboriginal health workers	9%	0%	2%	5%	5%	5%
Nurses	25%	2%	3%	17%	33%	17%*
Midwives	40%	82%	50%	25%	28%	39%*
Doctors	10%	16%	13%	51%	3%	25%*
Unknown	16%	0%	32%	2%	31%	14%*
Proportion of women with folic acid prescribed before 20 weeks	29%	49%	3%	33%	24%	27%*
Proportion of women with folic acid prescribed prior to conception	12%	7%	0%	8%	16%	8%
Proportion of women with iron prescribed	76%	73%	29%	63%	41%	58%*
Proportion of women with a general antenatal care plan present in the file	93%	98%	65%	82%	86%	83%

* P < 0.05 for comparisons between regions.

Across services the median age of women was 25.3 years (range 14-48) (Table 1). Compared with services in the NT, WA and North QLD, Far West NSW had a higher proportion of non-Indigenous women presenting for antenatal or postnatal care (P < 0.05), however, overall 89% of all women were Indigenous.

Across services, the proportion of women presenting for their first antenatal visit in the first trimester ranged from 34% to 49% and the mean gestational age at first visit was 16 weeks (range 3-40). The first antenatal assessment was undertaken by a variety of providers; with the exception of WA and North QLD services, the most common provider was a midwife (Table 1).

Documentation of prescription of folic acid was poor. Across all services only 27% of women were prescribed folic acid prior to 20 weeks gestation and even fewer (8%) prior to conception. In contrast, more than half of women in most services were prescribed iron during pregnancy. Over 80% of women had an antenatal care plan recorded in their file (Table 1).

The mean gestational age at birth was 38.6 weeks, and the majority of women had a vaginal birth (67%) (Table 2). The proportion of women who gave birth to an infant weighing < 2500 grams ranged from 5-15% across services.

**Table 2:** Birth outcomes

	NT Top End	NT Central Australia	Far West NSW	WA	North QLD	Total
**No. of health centres | client records**	**13 | 136**	**2 | 45**	**6 | 103**	**9 | 193**	**4 | 58**	**34 | 535**

Mean gestational age at birth (weeks)	38.8	38.8	38.8	38.4	38.3	38.6
Mean infant birth weight (grams)^†^	3203	3391	3415	3101	2995	3198*
Proportion of infants < 2500 grams at birth^†^	5%	7%	5%	14%	15%	10%
Proportion of infants < 37 weeks gestation	6%	11%	7%	14%	15%	11%
Type of birth						
Vaginal	64%	69%	60%	77%	52%	67%
Caesarean section	25%	29%	23%	22%	8%	22%*
Unknown	11%	2%	17%	1%	40%	11%*

† Birth weights were known for 468 infants, which accounted for 87% of the whole sample.

* P < 0.05 for comparisons between regions.

Recorded use of cigarettes at any time in the pregnancy was high across all services (43% in total), followed by use of alcohol (22%) and illicit drugs (9%) (Table 3). Among those who used cigarettes, 46% had documented advice regarding smoking cessation. Just over a third of all women had documented medical risk factors, and one-fifth had recorded social risk factors (e.g. related to domestic/social environment, finances or family/social support). The proportion of these risk factors was significantly different across regions. Documentation of brief interventions/advice regarding health behaviour/risks varied; 52% of women received antenatal education, whilst enquiries and advice around housing conditions, financial security and food security were the least frequently recorded (range, 6-11% for all women).

**Table 3:** Recording of pregnancy risk factors and brief interventions and delivery of routine antenatal checks

Item	NT Top End	NT Central Australia	Far West NSW	WA	North QLD	Total
**No. of health centres | client records**	**13 | 136**	**2 | 45**	**6 | 103**	**9 | 193**	**4 | 58**	**34 | 535**

**Any use of: **Cigarettes	41%	40%	39%	42%	55%	43%^†^
Alcohol	12%	27%	19%	25%	31%	22%*
Illicit drugs	7%	2%	17%	8%	7%	9%
1^st ^trimester: Cigarettes	32%	27%	24%	32%	43%	31%
Alcohol	9%	24%	13%	18%	28%	16%
Illicit drugs	5%	2%	9%	6%	3%	6%
3^rd ^trimester: Cigarettes	21%	38%	35%	26%	50%	30%
Alcohol	4%	13%	17%	14%	21%	13%
Illicit drugs	3%	0%	15%	6%	7%	6%
**Evidence of: **Social risk factors	11%	29%	6%	22%	57%	20%*
Medical risk factors	38%	64%	19%	39%	45%	38%*
**Brief interventions/counselling**						
Smoking cessation^‡^	48%	67%	35%	49%	41%	46%
Antenatal education	51%	93%	51%	46%	47%	52%*
Nutrition	53%	76%	18%	32%	59%	41%*
Oral health	33%	29%	5%	9%	7%	16%*
Breast feeding	21%	51%	17%	25%	19%	24%
Alcohol and other substance abuse	37%	56%	12%	39%	34%	34%*
Physical activity	27%	36%	5%	17%	38%	21%*
Mood (depression)	14%	51%	11%	20%	19%	19%*
Domestic/social environment	24%	58%	10%	16%	29%	22%
Social/family support	38%	73%	5%	29%	24%	30%*
Financial situation	2%	29%	1%	6%	7%	6%*
Housing condition	4%	44%	1%	15%	9%	11%*
Food security	2%	33%	1%	8%	7%	7%*
**Routine antenatal checks**						
Before 13 weeks gestation						
Weight	55%	56%	26%	33%	38%	40%
Maternal height	18%	29%	15%	19%	26%	20%
BMI	6%	27%	2%	14%	7%	10%*
BP	57%	56%	33%	38%	50%	45%
Urinalysis	57%	53%	24%	34%	34%	40%*
Between 13 and 24 weeks gestation						
Fundal height	80%	80%	36%	58%	67%	62%*
FHR	76%	78%	45%	57%	57%	61%*
BP	80%	82%	54%	67%	83%	71%
Urinalysis	79%	76%	29%	61%	59%	61%*
After 24 weeks gestation						
Fundal height	93%	89%	66%	69%	64%	76%*
FHR	93%	89%	70%	69%	60%	76%*
BP	93%	91%	71%	75%	78%	81%*
Urinalysis	95%	89%	52%	68%	57%	72%*

BMI = body mass index, FHR = fetal heart rate, BP = blood pressure.

* P < 0.05 for comparisons between regions.

† The reason that the proportion of women smoking at any time in pregnancy is higher than in either the first or third trimester is that of the 228 (43%) who had smoked at any stage during pregnancy, 101 smoked in both 1^st ^and 3^rd ^trimesters, 66 smoked only in 1^st ^trimester, and 61 smoked only in 3^rd ^trimester. Thus a proportion of women appear to have taken up smoking between the 1^st ^and 3^rd ^trimesters and a proportion who smoked in the first trimester were reported to no longer smoke in the 3^rd ^trimester.

‡ Among those who used cigarettes: NT Top End (n = 56); NT Central Aust (n = 18); Far West NSW (n = 40); WA (n = 82); North Qld (n = 32); total N = 228.

Consistent with the late gestational age at first presentation observed across services, documentation of routine antenatal checks including maternal height/weight, blood pressure and urinalysis before 13 weeks gestation was poor (range 10-45% for all women), but improved for checks undertaken between 13-24 weeks gestation (range 61-71% for all women), and after 24 weeks gestation (range 72-81% for all women) (Table 3). There was significant variation between regions in the frequency of routine antenatal checks.

Recording of routine laboratory investigations was high for most investigations undertaken at the first antenatal visit (range 63-82% for all women) (Table 4), with the exception of fetal anomaly screening (either nuchal translucency, first trimester combined screen or maternal serum screening, 15% for all women).

**Table 4:** Delivery of laboratory and ultrasound investigations

Service item	NT Top End	NT Central Australia	Far West NSW	WA	North QLD	Total
**No. of health centres | client records**	**13 | 136**	**2 | 45**	**6 | 103**	**9 | 193**	**4 | 58**	**34 | 535**

**First antenatal assessment**						
Blood group/Rhesus	96%	100%	65%	77%	79%	82%*
Antibodies	93%	100%	66%	70%	78%	79%*
MSU	91%	96%	40%	67%	76%	71%*
FBE	95%	100%	64%	73%	79%	80%*
Rubella	92%	100%	61%	70%	78%	77%*
HBsAg	91%	100%	56%	75%	79%	78%*
Syphilis serology	94%	100%	58%	55%	81%	72%*
HIV	80%	89%	14%	72%	59%	63%*
Offered fetal anomaly screening	6%	33%	17%	20%	0%	15%*
**Fetal anomaly screening**						
Client agreed to fetal anomaly screening	3%	18%	18%	15%	0%	11%*
Nuchal translucency	0%	13%	6%	3%	0%	3%*
First trimester combined screen	0%	11%	10%	3%	2%	4%*
Maternal serum screening 14-20 weeks	2%	2%	17%	8%	2%	7%*
**Investigations between 20 and 28 weeks**						
50 or 70 g glucose challenge test or glucose tolerance test	78%	49%	33%	38%	66%	51%*
FBE	82%	69%	24%	46%	60%	54%*
**Investigation between 34 and 37 weeks**						
Low vaginal swab for GBS	49%	62%	31%	29%	10%	35%*
**Routine ultrasound check**						
Before 16 weeks	32%	49%	38%	39%	24%	36%
16-20 weeks	47%	69%	31%	41%	34%	42%

FBE = full blood examination, GBS = group B streptococcus, HBsAg = hepatitis B antigen, HIV = human immunodeficiency virus, MSU = midstream urine.

* P < 0.05 for comparisons between regions.

Documentation of routine ultrasound varied between regions. Overall only 42% of women had a recorded ultrasound between 16-20 weeks gestation for fetal morphology (range 31-69% across regions) (Table 4). Similarly documentation of screening or diagnostic tests for gestational diabetes mellitus (GDM) varied (51% for all women, range 33-78%) as did investigation for Group B Streptococcus (GBS) infection (35% for all women, range 10-62%).

Documentation of follow up of identified problems varied (Table 5). Amongst women with an abnormal BP reading either before or after 28 weeks gestation, over 70% were referred to and examined by a GP or obstetrician and had follow up BP measurements. Similarly, amongst women with an abnormal screening test for GDM, 77% had a diagnostic oral glucose tolerance test. The proportion of women with a Rhesus negative blood group was low (3%), and compliance with anti-D injections varied depending on the recommended timing of the injection in the antenatal (range 40-50% for all women) and postnatal period (range 0-100%). Less than a third of all women with inadequate immunity had rubella immunisation documented postnatally.

**Table 5:** Response to abnormal clinical findings

	NT Top End	NT Central Australia	Far West NSW	WA	North QLD	Total
**No. of health centres | client records**	**13 | 136**	**2 | 45**	**6 | 103**	**9 | 193**	**4 | 58**	**34 | 535**

**Record of abnormal BMI (< 20 or > 30)**	3% (4/136)	20% (9/45)	2% (2/103)	6% (12/193)	5% (3/58)	6% (30/535)*
Documented plan of management	0% (0/4)	44% (4/9)	0% (0/2)	0% (0/12)	0% (0/3)	13% (4/30)*
**Record of abnormal BP (≥140/90) prior to 28 weeks**	1% (2/136)	11% (5/45)	1% (1/103)	0% (0/193)	0% (0/58)	2% (8/535)*
Follow up BP done	100% (2/2)	60% (3/5)	100% (1/1)	n/a	n/a	75% (6/8)
Urine tested for protein	100% (2/2)	60% (3/5)	100% (1/1)	n/a	n/a	75% (6/8)
GP/Obstetric referral	50% (1/2)	80% (4/5)	100% (1/1)	n/a	n/a	75% (6/8)
Examination by GP/Obstetrician	50% (1/2)	80% (4/5)	100% (1/1)	n/a	n/a	75% (6/8)
Anti-hypertensive medication prescribed	50% (1/2)	20% (1/5)	100% (1/1)	n/a	n/a	38% (3/8)
**Record of abnormal BP (≥140/90) at or after 28 weeks**	1% (1/136)	13% (6/45)	5% (5/103)	3% (6/193)	0% (0/58)	3% (18/535)*
Follow up BP done	100% (1/1)	100% (6/6)	60% (3/5)	50% (3/6)	n/a	72% (13/18)
Urine tested for protein	100% (1/1)	100% (6/6)	60% (3/5)	50% (3/6)	n/a	72% (13/18)
GP/Obstetric referral	100% (1/1)	100% (6/6)	60% (3/5)	50% (3/6)	n/a	72% (13/18)
Examination by GP/Obstetrician	100% (1/1)	100% (6/6)	100% (5/5)	50% (3/6)	n/a	83% (15/18)
Anti-hypertensive medication prescribed	100% (1/1)	33% (2/6)	0% (0/5)	17% (1/6)	n/a	22% (4/18)
**Record of abnormal standard glucose challenge test**	17% (23/136)	22% (10/45)	10% (10/103)	4% (7/193)	17% (10/58)	11% (60/535)*
GTT undertaken	87% (20/23)	90% (9/10)	80% (8/10)	43% (3/7)	60% (6/10)	77% (46/60)
**Rhesus negative**	1% (1/136)	0% (0/45)	7% (7/103)	4% (8/193)	3% (2/58)	3% (18/535)*
Anti-D injection given 26-28 wks	100% (1/1)	n/a	43% (3/7)	63% (5/8)	0% (0/2)	50% (9/18)
Anti-D injection given 34-36 wks	100% (1/1)	n/a	29% (2/7)	63% (5/8)	0% (0/2)	40% (8/18)
**Baby Rhesus positive**	0% (0/136)	33% (15/45)	2% (2/103)	1% (2/193)	0% (0/58)	4% (19/535)*
Mother given anti-D postnatal	n/a	0% (0/15)	100% (2/2)	100% (2/2)	n/a	21% (4/19)*
**Anaemia (Hb < 100 g/L)**	14% (19/136)	22% (10/45)	11% (11/103)	12% (24/193)	3% (2/58)	12% (66/535)*
Iron prescribed	84% (16/19)	100%(10/10)	91% (10/11)	75% (18/24)	50% (1/2)	83% (55/66)
Follow up FBE or Hb test done	42% (8/19)	90% (9/10)	36% (4/11)	46% (11/24)	50% (1/2)	50% (33/66)
**Nitrites detected by dip stick**	21% (28/136)	33% (15/45)	5% (5/103)	24% (46/193)	10% (6/58)	19% (100/535)*
Urine sent for MSU	96% (27/28)	100%(15/15)	100% (5/5)	93% (43/46)	100% (6/6)	96% (96/100)
Oral antibiotic prescribed	93% (26/28)	60% (9/15)	80% (4/5)	37% (17/46)	83% (5/6)	61% (61/100)*
Record of a normal follow up MSU	46% (13/28)	100%(15/15)	40% (2/5)	26% (12/46)	83% (5/6)	47% (47/100)*
**Rubella antibodies negative or low titre**	35% (47/136)	7% (3/45)	15% (15/103)	15% (28/193)	7% (4/58)	18% (97/535)*
Rubella immunisation given postnatally	36% (17/47)	67% (2/3)	13% (2/15)	32% (9/28)	0% (0/4)	31% (30/97)

BP = blood pressure, BMI = body mass index, FBE = full blood examination, GP = general practitioner, GTT = glucose tolerance test, Hb = haemoglobin, MSU = midstream urine, n/a = not applicable.

* P < 0.05 for comparisons between regions.

Although 53% of women had a recorded postnatal visit, documentation of advice regarding health risk factors was poor (Table 6). For around half of all women there was documentation about breastfeeding advice and contraception. However advice about smoking, nutrition and mood (depression) were infrequently recorded (range 19-21% for all women), as was advice around sudden infant death syndrome (SIDS) prevention, injury prevention and infection/hygiene (range 4-5% for all women).

**Table 6:** Recording of brief interventions after birth

	NT Top End	NT Central Australia	Far West NSW	WA	North QLD	Total
**No. of health centres | client records**	**13 | 136**	**2 | 45**	**6 | 103**	**9 | 193**	**4 | 58**	**34 | 535**

**Recorded postnatal visit**	55%	87%	65%	41%	36%	53%*
**Brief interventions/counselling**						
Smoking	12%	27%	25%	17%	24%	19%
Nutrition	13%	42%	26%	15%	34%	21%*
Breast feeding	37%	82%	65%	42%	31%	47%*
Infection prevention/hygiene	7%	7%	3%	5%	5%	5%
Injury prevention	1%	0%	9%	4%	2%	4%
SIDS prevention	1%	2%	17%	4%	0%	5%*
Alcohol and other substance abuse	5%	9%	21%	15%	16%	13%*
Physical activity	5%	20%	21%	9%	19%	12%*
Mood (depression)	13%	33%	31%	19%	7%	20%*
Contraception	41%	82%	56%	34%	24%	43%*
Domestic/social environment	8%	22%	7%	11%	10%	10%*
Social/family support	12%	44%	20%	19%	9%	19%*
Financial situation	3%	7%	3%	9%	2%	5%
Housing condition	1%	20%	1%	15%	3%	8%*
Food security	1%	16%	0%	6%	2%	4%*

SIDS = sudden infant death syndrome.

* P < 0.05 for comparisons between regions.

## Discussion

The clinical audit data presented here provide insights into documented delivery of maternity care which should be useful for informing local, regional and national efforts to promote the quality of primary maternal health care for Indigenous women. Participating services varied in their relative strengths and weaknesses, both geographically and between service items. Nevertheless, adherence to recommended screening investigations and brief interventions/advice about health behaviours were consistently identified as areas for improvement across services.

This study extends previous work demonstrating improvements in antenatal screening at a local level [14,15] and indicates the potential for improvements to be replicated on a broad scale. The study also demonstrates the potential for quality improvement methods to be extended to systematic assessment of other aspects of care including follow up of abnormal clinical findings. In addition, this study adds to the limited international literature on approaches to assessing the quality of antenatal care in primary care settings.

Overall, there was good documentation around routine checks in pregnancy such as blood pressure, and in the follow up of abnormal clinical findings related to hypertension and GDM, where rates of specialist examination were high. Wide variations were present in documentation of routine investigations such as morphology ultrasound and screening tests for GDM and GBS. On average documentation of these procedures was only around 40-50%, despite evidence for treatment of carbohydrate intolerance in pregnancy [26] and GBS screening [27].

Across all services, prescription of peri-conceptional folic acid supplements was low. These findings may reflect later presentation for care in pregnancy and indicate that fortified food is a potentially important source of folate to protect against neural tube defects (NTD) in those communities. Importantly, Australia recently introduced a policy of mandatory fortification of certain foods with folic acid for additional protection against NTD.

Uptake of fetal anomaly screening was low, which supports previous calls for the development of culturally safe resources around these tests [13]. There is considerable room for improvement in other aspects of antenatal counselling and education. Good evidence exists for the effectiveness of smoking cessation advice in pregnancy [28], yet documentation of this information was present for only 46% of smokers, despite the high proportion of women with recorded use of cigarettes. There was virtually no change in the proportion of women who smoked in the first compared to the third trimester overall, although in two regions the proportion declined (Top End 32%-21%; WA 32%-26%) (Table 3). Cessation advice that is inappropriate to the cultural context may be a barrier to addressing smoking in pregnancy [29] and we await the results of a controlled trial of a culturally-specific smoking intervention for pregnant Indigenous women [30]. It should be noted that many mainstream maternity units in Australia do not have relevant smoking cessation protocols [31].

In the postnatal period, only 55% of women received breastfeeding advice, and there was poor documentation of advice around smoking, hygiene, injury prevention and SIDS prevention. This is a significant area for improvement given the high prevalence of SIDS risk factors such as smoking, preterm birth and low birth-weight in these communities. Overall, this indicates there is great potential to improve the continuity of care throughout the antenatal and postnatal period. It should be noted that centres in this study do not provide birthing services; local policies currently require women in remote areas to be transferred into regional hospitals for birthing from 36 weeks gestation. Therefore, attempts to improve continuity in this setting will require better integration of services or a redesign of care to support community-based services across the spectrum of maternity care. This was acknowledged in the recent report of the Australian Maternity Services Review [32], which also recognised the importance of provision of services to Indigenous women that accommodate cultural beliefs about childbirth, including in some communities, a preference for 'birthing on country'.

The findings of this study are subject to some limitations. Participating services have a history of involvement in quality improvement activities, expanded from a focus on chronic disease management to maternal, child and other aspects of health, which may limit the generalisability of findings. The maternal audit was undertaken to provide baseline data for individual services with an interest in improving delivery of maternal health care, as part of an on-going quality improvement study (the ABCD project). Not all services participating in this larger study elected to audit maternal records, which may also limit the generalisability of findings. Our audit data provide similar estimates of mean maternal age and smoking prevalence, but lower estimates of preterm birth and low birth weight than estimates reported in routine NT, WA, QLD and NSW Indigenous perinatal information [2]. As such, our data should not be regarded as representative of all services in the regions. Furthermore, small numbers of abnormal findings in this sample means estimates of rates of follow up of abnormal findings lack precision. The data presented here are baseline service activity only; more data will be available for subsequent years of the quality improvement intervention, which includes annual cycles of organisational assessment, clinical audits, feedback to and interpretation of data with participating health centre staff, action planning, goal setting and implementing of strategies for change [18].

The measures of quality of care reported in this study provide important data for feedback to services at a local level. When aggregated, they provide a picture of regional patterns of maternity care which will be progressively more informative for broad scale policy and program purposes as increasing numbers of services become involved. Importantly, these measures provide information that is not currently available from routinely collected perinatal data [2].

There is increasing activity in Australia and internationally around assessment of maternity care, with the completion several projects designed to facilitate benchmarking of services [33-35]. The two recent Australian projects recommended a core set of indicators focussing on intrapartum and postpartum clinical outcomes. In developing our study we had difficulty choosing measures of quality of maternal health care relevant for the community health centre setting. Reporting on the proposed national maternity indicators would require information from other systems such as hospital administrative data. It is also unrealistic to expect to show improvements in outcomes such as low birth weight over annual cycles of quality improvement activities. We chose to focus on processes of antenatal and postnatal care, which reflect delivery of routine screening investigations and treatment of abnormal findings, consistent with local guidelines for the community health setting. We recognise that like the proposed national indicators, this presents an incomplete picture of the scope of maternity care. A coordinated and consistent national approach to reporting of maternity information across all services (primary, secondary and tertiary) is needed, building on the substantial work of the National Perinatal Statistics Unit within the Australian Institute of Health and Welfare in national perinatal data development. Achieving this will require consensus on standard components of antenatal care, and so it is critical for Governments to continue to support the development of national guidelines defining best practice in antenatal care in Australia, such as those in the United Kingdom [36].

## Conclusion

Supporting services to improve maternity care for Indigenous women is a recognised priority [16] given the persistence of unacceptably high rates of poor Indigenous perinatal outcomes. This is acknowledged by Indigenous women, who consistently identify optimising the health of mothers and babies as a high priority [21,37,38]. This study demonstrates that although Indigenous women presented at a later gestational age they regularly attended services for maternity care, with the average total number of antenatal visits within suggested minimum numbers [5,20]. Importantly, this indicates that there are opportunities for appropriate investigation and risk factor intervention amongst Indigenous women. The findings highlight the inadequacy of existing systems in community health centres to provide access to routine evidence-based screening and health information in the antenatal and postnatal period. More broadly, the findings reflect the lack of consistency in national standards defining antenatal care that all Australian women should expect to receive, which unless addressed, will see the persistence of inequalities in maternity care and outcomes.

## List of abbreviations used

ABCD: Audit and Best practice for Chronic Disease; BMI: Body Mass Index; BP: Blood Pressure; FBE: Full Blood Examination; FHR: Fetal Heart Rate; GBS: Group B Streptococcus; GDM: Gestational diabetes mellitus; GP: General Practitioner; GTT: Glucose Tolerance Test; Hb: Haemoglobin; HBsAg: Hepatitis B Antigen; HIV: Human Immunodeficiency Virus; MSU: Midstream Urine; NSW: New South Wales; NT: Northern Territory; NTD: Neural Tube Defects; QLD: Queensland; Rh: Rhesus; SIDS: Sudden Infant Death Syndrome; WA: Western Australia; WBM: Women's Business Manual.

## Competing interests

The authors declare that they have no competing interests.

## Authors' contributions

ARR played a primary role in the interpretation of study results and preparation of the manuscript. RSB developed the study design, managed the implementation of the study, and played a primary role in the interpretation of study results and preparation of the manuscript. DS was responsible for data management and data analysis and contributed to preparation of the manuscript. MCD, RJC, LO, HEL, RKK, SCT, HPB, ADHB, TW, CMC provided expert advice in contextualising study findings, particularly in relation to each jurisdiction, and assisted with preparation and editing of the manuscript. All authors read and approved the final manuscript.

## Pre-publication history

The pre-publication history for this paper can be accessed here:

http://www.biomedcentral.com/1471-2393/11/16/prepub
